# Emerging Role of Ferroptosis in Acute Kidney Injury

**DOI:** 10.1155/2019/8010614

**Published:** 2019-10-31

**Authors:** Zhaoxin Hu, Hao Zhang, Shi-kun Yang, Xueqin Wu, Dong He, Ke Cao, Wei Zhang

**Affiliations:** ^1^Department of Nephrology, The Third Xiangya Hospital, Central South University, Changsha, 410013 Hunan Province, China; ^2^Department of Respiration, The Second People's Hospital of Hunan Province of Hunan University of Chinese Medicine, Changsha, Hunan, China; ^3^Department of Oncology, The Third Xiangya Hospital, Central South University, Changsha, 410013 Hunan Province, China

## Abstract

Acute kidney injury (AKI) is a heterogeneous group of critical disease conditions with high incidence and mortality. Vasoconstriction, oxidative stress, apoptosis, and inflammation are generally thought to be the main pathogenic mechanisms of AKI. Ferroptosis is a type of iron-dependent nonapoptotic cell death characterized by membrane lipid peroxide accumulation and polyunsaturated fatty acid consumption, and it plays essential roles in many diseases, including cancers and neurologic diseases. Recent studies have revealed an emerging role of ferroptosis in the pathophysiological processes of AKI. Here, in the present review, we summarized the most recent discoveries on the role of ferroptosis in the pathogenesis of AKI as well as its therapeutic potential in AKI.

## 1. Introduction

**Figure 1:**
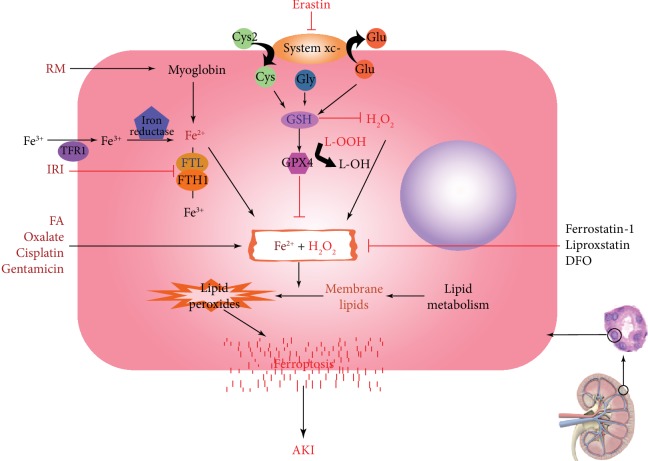
In renal tubular cells, abnormal increases in Fe^2+^ or H_2_O_2_ caused by various pathogenic factors trigger the Fenton-like chemistry, which oxidizes membrane lipids to lipid peroxides, and mediate ferroptosis thus leading to AKI. RM initiates ferroptosis by raising the level of Fe^2+^, while IRI induces ferroptosis by inhibiting the conversion of Fe^2+^ to Fe^3+^. Other pathogenic factors such as FA, oxalate, cisplatin, and gentamicin can also promote the occurrence of Fenton-like chemistry, induce ferroptosis, and lead to AKI. The Fenton-like chemistry can be inhibited by intracellular GPX4, a key enzyme that maintains tissue homeostasis. Ferrostatin-1, liproxstatin, and DFO alleviate or delay the development of AKI by inhibiting the Fenton-like chemistry in a protective role in ferroptosis induced by various pathogenic factors. Note: AKI: acute kidney injury; RM: rhabdomyolysis; IRI: ischemia reperfusion injury; FA: folic acid; GPX4: glutathione peroxidase 4; DFO: deferoxamine.

Acute kidney injury (AKI), formerly known as acute renal failure (ARF), is a common and critical illness caused by multiple causes, including ischemia, nephrotoxic drugs, and urinary tract obstruction [1]. AKI occurs in approximately 5% of hospitalized patients and 30% of critically ill patients and has high morbidity and mortality [2]. Moreover, studies have shown that AKI increases the potential risk of chronic kidney disease and end-stage renal disease in patients [3–5]. In addition to blood purification, few therapeutics have made significant progress in the prevention of AKI. Thus, new targets or better regimens are still urgently needed to prevent AKI as well as to facilitate adaptive repair after the occurrence of AKI. The pathogenesis of AKI was previously believed to involve vasoconstriction, oxidative stress, apoptosis, inflammation, and hypoxia [6]. In 2012, Dixon et al. proposed a new concept of cell death, namely, ferroptosis [7], which was subsequently demonstrated to be involved in diseases such as cancers and in neurological disorders including Huntington disease and periventricular leukomalacia [8, 9]. A quite recent study shows that Hepcidin, a major regulator of iron homeostasis, plays a protective role in AKI. This also provided a new evidence of the role of iron homeostasis in the pathogenesis of AKI and the therapeutic potential for AKI [10]. This review summarizes the current research progress on ferroptosis, its regulatory mechanisms, and its therapeutic potential in AKI (see graphic summary in Figure 1).

## 2. Definition, Process, and Measurement of Ferroptosis

Ferroptosis is iron-dependent nonapoptotic cell death and is characterized by the accumulation of membrane lipid peroxidation products and the consumption of plasma membrane polyunsaturated fatty acids. This kind of cell death can be induced by specific small molecules such as erastin and RAS-selective lethal 3 (RSL3) [11]. Ferroptosis has been reported to participate in various pathological processes of the brain, kidney, liver, and heart diseases [12]. In many cells, the import of cystine (Cys2) through system xc- is required for glutathione (gL-glutamyl-L-cysteinylglycine [GSH]) synthesis and maintains the function of glutathione peroxidase 4 (GPX4) [13]. Erastin (a ferroptosis inducer) can inhibit the import of Cys2, leading to GSH depletion and inactivation of phospholipid peroxidase and GPX4 [14, 15]. GSH depletion can lead to iron-dependent accumulation of reactive oxygen species (ROS), especially lipid ROS, which are themselves sufficient to kill cells [7].

Iron metabolism and lipid peroxidation signaling are thought to be central mediators of ferroptosis [16]. Circulating iron exists in the form of ferric iron (Fe^3+^) bound to transferrin. Fe^3+^ is introduced into the cell via the membrane protein transferrin receptor 1 (TFR1) and then localized to the endosome. In the endosomes, iron reductase reduces Fe^3+^ to ferrous iron (Fe^2+^). Finally, divalent metal transporter 1 (DMT1) mediates the release of Fe^2+^ from the endosomes into unstable iron pools in the cytoplasm. Excessive iron is stored in ferritin, an iron storage protein complex that includes ferritin light chain (FTL) and ferritin heavy chain 1 (FTH1) [17]. FTH has iron oxidase activity, which catalyzes the conversion of the ferrous form (Fe^2+^) to the ferric form (Fe^3+^), allowing iron to be safely incorporated into the ferritin shell, thereby reducing free iron levels [18]. Hydrogen peroxide (H_2_O_2_) can react with ferrous ions and produce hydroxyl radicals with strong oxidizing properties, this reaction is called Fenton reaction [19]. Excessive iron can lead to the production of ROS that mediate ferroptosis through Fenton-like chemistry [20].

Compared with other forms of regulatory cell deaths, such as apoptosis and autophagy, ferroptosis has unique morphological characteristics and biological manifestations. When ferroptosis occurs, the cell membrane ruptures and get blistered, the cell nucleus become lacking of chromatin condensation, the mitochondria decreases, mitochondria size decreases, the density of the bilayer membrane increases, the mitochondrial cristae decrease or disappear, and the mitochondrial outer membrane ruptures as observed under electron microscopy [17, 21]. However, whether mitochondrial damage plays a role in ferroptosis or just represents its irreversible result remains controversial [22]. The biological properties of ferroptosis are characterized by iron and ROS aggregation, which inhibit the activities of system xc- and GPX4 by reducing cystine uptake, depleting GSH, and releasing arachidonic acid and other molecules [13]. This process is different from neither apoptosis nor autophagy (see brief comparison in Table 1).

Alone with the characteristics of ferroptosis described above, we usually assess ferroptotic cell death by cell death staining like TUNEL assay [17]. At the same time, we can measure the levels of Fe^2+^, 4-hydroxynonenal (4HNE), and malondialdehyde (MDA) in tissues or cell lysate to assess iron-related lipid peroxidation [23, 24]. In addition, GPX4 and GSH levels should be tested to assess the inhibition of antioxidants during ferroptosis [24, 25]. We can further support ferroptosis by observing mitochondrial atrophy and rupture of the adventitia under transmission electron microscopy [26]. In another way, the application of ferroptosis inhibitors like ferrostatin-1 and liproxstatin-1 was also a necessary method to observe and demonstrate the occurrence of ferroptosis.

## 3. Key Regulators of Ferroptosis

### 3.1. GPX4

GPX4 was first isolated and purified from pig liver in 1982 by Ursini and colleagues, and its ability to inhibit iron-catalyzed lipid peroxidation in microsomes has been proven [15, 27]. GPX4 is one of the eight well-known GSH peroxidases in mammals. It is the only enzyme capable of reducing esterified oxidized fatty acids and cholesterol hydroperoxides [28], reducing lipid hydroperoxide (L-OOH) to a nontoxic lipid hydroxy derivative (L-OH). GSH is an essential intracellular antioxidant synthesized by glutamic acid (Glu), cysteine (Cys), and glycine (Gly) [29]. It can be used for enzymatic and nonenzymatic antioxidant reactions in cells and can maintain hydrogen peroxide levels within the physiological range. Cellular GSH levels are not depleted under normal physiological conditions [30]. GPX4 is a central enzyme that utilizes GSH to counteract lipoxygenase (Alox) activity and phospholipid/cardiolipin oxidation events, thus reducing peroxides at the expense of GSH or other thiol-containing compounds [31]. GPX4 is essential for maintaining tissue homeostasis and prevents cell death and tissue damage in multiple organs, including the brain, skin, and endothelium [32]. RSL3 induces ferroptotic cell death by binding and inactivating GPX4 [14]. GPX4 knockout in tubular cells results in massive cell deaths associated with the pathological form of ferroptosis [33]. In addition, sensitivity profiling in 177 cancer cell lines revealed that diffuse large B cell lymphomas and renal cell carcinomas were particularly susceptible to GPX4-regulated ferroptosis [14]. All of the above observations confirmed a negative regulation of ferroptosis by GPX4.

### 3.2. System xc-

System xc- is thought to be another important regulator of ferroptosis. It is the cystine/glutamate reverse transporter and consists of two subunits, xCT and 4F2hc. System xc- is one of many amino acid transporters expressed on the plasma membrane of mammalian cells, and it exchanges intracellular glutamate with extracellular cystine at a molar ratio of 1 : 1 [34]. Cystine is rapidly reduced to cysteine by systemic xc- uptake, which is used to synthesize proteins and GSH—the main endogenous antioxidant in mammalian cells [35]. Dietary cystine uptake and reabsorption in the kidney are important to maintain the required amino acid levels in the body. Early studies on monkeys have confirmed that xCT are mostly located at the brush border membrane of renal tubules, which is the anatomical location of amino acid transport in the kidney. It is important to emphasize that specific in-house developed antibodies were used to detect xCT expression in the kidney in this study [30]. Inhibition of system xc- can lead to a rapid decline in GSH levels and a rapid death of a variety of cell types *in vitro* [36]. Studies have found that ferroptosis induced by erastin was similar in many aspects to sulfasalazine- (SAS-) induced cell death, and SAS is a well-known system xc- inhibitor [7, 37]. Erastin, SAS, and sorafenib can block the uptake of radiolabeled cystine in cancer cells in culture [38]. Therefore, erastin appears to act as a direct inhibitor of system xc- function, suggesting that erastin induces ferroptosis through inhibiting system xc-. In addition, Beclin1 (BECN1), which is a key regulator of macroautophagy/autophagy and is involved in the production of the phosphatidylinositol 3-kinase (PtdIns3K) complex, was recently found to be a new driver of ferroptosis in a study showing that BECN1 may promote ferroptosis by regulating the activity of system xc- in cancer cells [39].

## 4. Ferroptosis and AKI

In geriatric and degenerative diseases, iron levels in the brain are inevitably elevated. The oxidative stress of excess iron is related to carcinogenesis [40–42], highlighting the involvement of ferroptosis. Recent findings have also revealed a link between ferroptosis and human diseases. The pathogenesis of AKI is very complicated, and the proximal tubule segment of the nephron is most susceptible to various forms of injury due to its anatomical features and complex functions [43]. A variety of molecular mechanisms have been proposed to induce or aggravate AKI, but ROS-induced renal damage is considered to be one of the key mediators [16, 44–46]. Multiple studies have suggested that ferroptosis is a promising therapeutic target, especially in diseases dominated by kidney tubular necrosis [47]. In a recent study using inducible GPX4-deficient mice, the mice died of massive renal tubular cell death and acute renal failure within 2 weeks after GPX4 deletion. The survival of the GPX4-deficient mice could be extended by approximately 35% by the clearance of lipid peroxides *in vivo* [33], further indicating an essential role of ferroptosis in kidney injury. More recently, studies on rhabdomyolysis and ischemia/reperfusion-induced AKI or other AKI models have provided additional direct evidence supporting the involvement of ferroptosis in AKI (Figure 1).

### 4.1. Ferroptosis and AKI Caused by Rhabdomyolysis

Rhabdomyolysis (RM) can be caused by strenuous exercise; direct trauma; and metabolic changes in the muscles; toxic effects of chemical, physical, or biological agents; and genetic factors [48–50]. Renal failure caused by RM accounts for 15% of all cases of acute renal failure [51]. Previous studies have suggested that the accumulation of myoglobin (Mb) in the kidney is the core mechanism leading to kidney damage. After lysis of myocytes, a large amount of salt, enzyme, and Mb are released in the circulation [52, 53], leading to circulating Mb deposition in the kidney which causes tubular obstruction and necrosis with strong renal vasoconstriction [54, 55]. Studies on RM-induced AKI indicate that Fe^2+^ produced by Mb metabolism directly induces lipid peroxidation in proximal tubular epithelial cells may be an important mechanism of RM-induced renal injury [56]. An animal model of myoglobinuria after intramuscular injection of glycerol is closely associated with human RM [57]. The free iron released by Mb degradation in the kidney participates in the production of oxidizing substances through the catalytic action of the Fenton reaction. Studies have shown that the use of the iron chelator deferoxamine can alleviate RM-induced renal injury in rats [58] and prevent direct exposure to Mb-induced cytotoxicity *in vitro* [59]. Guerrero-Hue et al. [60] demonstrated a key role for ferroptosis in RM-induced AKI and showed that ferroptosis-sensitive cell death can be inhibited by curcumin, a powerful antioxidant. Moreover, Zarjou et al. have reported that FTH knockout mice showed higher mortality and more severe kidney damage than the wild-type mice in RM-induced AKI model, indicating the protective effect of heavy chain ferritin against renal tubular injury and the role of iron ions in AKI [61]. Together, these studies have strongly suggested that ferroptosis plays an important role in RM-induced AKI.

### 4.2. Ferroptosis and AKI Induced by Ischemia/Reperfusion

Ischemia reperfusion injury (IRI) is characterized by a sudden pause in the blood supply to a particular organ and reoxygenation after restoration of blood flow. This process can exacerbate tissue damage by triggering an inflammatory cascade involving ROS, cytokines, chemokines, and leukocyte activation [62, 63]. In the kidney, IRI is one of the main causes of AKI. The pathophysiology of IRI in the kidney includes inflammation, oxidative stress and lipid peroxidation, mitochondrial dysfunction, renin-angiotensin system activation, and nitrite and nitric oxide accumulation [64]. In the past, apoptosis was considered the main regulated cell death in various models of ischemic injury. When necrotic apoptosis was found as a form of receptor interacting serine/threonine kinase 3 (RIPK3) and mixed lineage kinase domain-like- (MLKL-) dependent regulatory necrosis, it was considered to be the leading cause of ischemic injury to the heart and kidney [65, 66]. However, later discovery suggested that ferroptosis might be a major driver of ischemic injury [67]. The application of ferrostatin in severe IRI models protected mice from functional acute renal failure and structural organ damage [68]; moreover, the RIPK1 inhibitor necrostatin-1 (Nec-1) did not protect the freshly isolated tubules from hypoxic injury. Clinically, renal ischemia-reperfusion injury is the main cause of AKI in patients that have undergone cardiac surgery, and studies have shown that low levels of intraoperative iron-binding proteins may reflect an impaired ability to rapidly process catalytic iron released during extracorporeal circulation, leading to kidney damage [69], which highlights the importance of iron homeostasis in human ischemia-reperfusion injury and indicates that ferroptosis might be a potential therapeutic target in cardiac surgery-associated kidney injury or IRI-induced AKI.

### 4.3. Ferroptosis in Folic Acid-Induced AKI

Drugs and poisons are also common causes of AKI. Folic acid (FA) can cause AKI in rodents [70, 71], at a certain dose FA may form a crystal in the renal lumen [72] and at high doses FA is also directly toxic to tubular epithelium [73]. Lipid peroxidation in the kidney was found in animal models of FA-AKI, and mice pretreated with the ferrostatin-1 had improved renal function and reduced tissue damage [74]. However, this effect was not observed by inhibiting necrotic apoptosis or interfering with apoptosis at the pharmacological or genetic level. Therefore, ferroptosis is considered to be the main pathway for regulatory necrosis in FA-induced AKI.

### 4.4. Ferroptosis and Other AKI Models

Cisplatin is a widely used anti-tumor drug, and nephrotoxicity is its main side effect. Baliga et al. reported in 1998 that experiments with cisplatin-induced cytotoxicity models *in vitro* and cisplatin-induced acute renal failure *in vivo* demonstrated that exposure to cisplatin resulted in a significant increase in bleomycin-detectable iron [23]. The use of deferoxamine provided significant functional (measured by blood urea nitrogen and creatinine) and histological protection against cisplatin-induced acute renal failure. In the proximal tubule, FtH knockout mice [61] had more severe kidney damage after cisplatin administration than did the control mice. These studies have indirectly indicated a role for ferroptosis in cisplatin-induced kidney injury. However, further experimental validation using specific ferroptosis inhibitors and featuring more phenotypic observations of ferroptosis is needed in future studies on cisplatin-induced AKI.

In addition, reactive oxygen metabolites and scavengers of iron chelators also provide protection against oxalate crystals or gentamicin-induced nephrotoxicity [68, 75]. tert-Butyl hydroperoxide (tBHP) injured freshly isolated proximal tubules in an Fe-dependent fashion and that effect was ameliorated by a lipophilic antioxidant, diphenyl-p-phenylenediamine (DPPD). Protection against tBHP toxicity by deferoxamine and DPPD was accompanied by substantial suppression of thiobarbituric acid reactive substances (TBARS) accumulation [76]. These studies have directly or indirectly supported an essential role of ferroptosis in renal tubular injury.

## 5. Treatment of AKI by Targeting Ferroptosis

Ferrostatin-1 (Fer-1) acts by a redox reaction to prevent membrane lipid damage and thereby inhibit cell death [77]. It has been demonstrated to be able to reduce lipid peroxides to alcohols (R-OOH→R-OH) and/or to intercept and scavenge lipid groups by hydrogen atom transfer or direct reduction (R-O→R-OH) [78]. Ferrostatin is frequently reported to attenuate lipid peroxidation-mediated tissue damage in various diseases, including acute kidney disease. Liproxstatin, another featured inhibitor of ferroptosis, is a potent spiroquinoxalinamine derivative found by high-throughput screening that inhibits ferroptosis by lipid peroxide clearance *in vivo* [79]. Liproxstatin-1 was reported to suppress ferroptosis in human renal proximal tubule epithelial cells, in Gpx4^−/−^ kidney, and in an IRI-induced tissue injury model [33]. In addition, lipid peroxidation inhibitors, such as lysyl oxidase (LOX) inhibitors, also inhibit ferroptosis [80]. Antioxidants and iron chelators (such as vitamin E and deferoxamine [DFO]) were also observed to inhibit ferroptosis by reducing iron availability [81] (commonly used inducers or inhibitors are summarized in Table 2). Most work on these ferroptosis inhibitors were performed in rodent AKI models or in *in vitro* experiments and lack clinical application potential. Future clinical work on AKI prevention or therapy targeting ferroptosis is needed.

## 6. Conclusion

Ferroptosis, which represents a unique form of regulated cell death, has been reported to be involved in the development of multiple cancers and neurologic diseases. An increasing number of studies have suggested a significant role of ferroptosis in acute kidney disease. In various AKI models, ferroptotic processes were observed and (or) inhibited by ferroptosis inhibitors, such as ferrostatin-1 and liproxstatin-1, to exert renal protection (Figure 1). The pathophysiologic processes of AKI are complicated by apoptosis, necrosis, and other forms of cell death. The extent to which ferroptosis is involved in AKI caused by different hazard factors remains largely unknown. In addition to the general controversial questions, such as the relationship between ferroptosis and iron, lipid peroxidation, oxidative stress injury, and mitochondrial autophagy also need to be further addressed in renal diseases. Ways in which we can benefit more from ferroptosis regulation to protect the kidneys from acute injury and even chronic impairment need to be considered. More comprehensive and in-depth research on ferroptosis in the field of renal diseases is needed to expand our knowledge and techniques against renal impairment to gain benefit in clinical outcomes in the future.

## Figures and Tables

**Table 1:** The differences between ferroptosis and other forms of cell death.

Cell death type	Ferroptosis	Apoptosis	Autophagy
Cell membrane	Rupture	Complete	Complete
Characteristic	Pathological cell death	Self-killing	Self-eating
Typical change	Iron-dependent lipid peroxidation	Caspase-dependent chromatin condensation	Autophagosome formation

**Table 2:** Inducers and inhibitors of ferroptosis.

Ferroptosis inducer	Mechanism of action	Ferroptosis inhibitor	Mechanism of action
Erastin	Suppress system xc-	Ferrostatin-1	Reduce lipid peroxides
Sulfasalazine	Suppress system xc-	Liproxstatin	Reduce lipid peroxides
RSL3	Suppress GPX4	Deferoxamine	Reduce free iron
Sorafenib	Suppress system xc-	Vitamin E	Reduce free iron
